# Epidemiology of Periprosthetic Fractures After Cementless Revision Total Hip Arthroplasty with Tapered, Fluted Stems at a Mid- to Long-Term Follow-Up

**DOI:** 10.3390/jcm14051468

**Published:** 2025-02-22

**Authors:** Oliver E. Bischel, Matthias K. Jung, Arnold J. Suda, Jörn B. Seeger, Paul M. Böhm

**Affiliations:** 1 BG Trauma Center at University of Heidelberg, Ludwig-Guttmann-Str. 13, 67 071 Ludwigshafen, Germany; matthias.jung@bgu-ludwigshafen.de; 2 Medical Faculty Mannheim, University of Heidelberg, Ludolf-Krehl-Str. 13-17, 68 167 Mannheim, Germany; arnoldsuda@yahoo.com; 3 Parc Clinic, Am Kaiserberg 2-4, 61 231 Bad Nauheim, Germany; joernseeger@gmx.net; 4 General Orthopedics, Pläntschweg 25, 81 247 Munich, Germany; info@ortho-boehm.de

**Keywords:** modular revision stem, tapered revision stem, revision THA, periprosthetic fracture, survivorship analysis

## Abstract

**Background:** Although tapered and fluted stems are frequently used in revision total hip arthroplasty (RTHA), major complications following the implantation of these implants, like periprosthetic fractures, are less investigated. As epidemiological data do not exist yet, the incidence of PPF in a mid- to long-term follow-up is unknown and potential risk factors have not been detected. **Methods:** Propensity score matching (PSA) of two retrospectively investigated cohorts after femoral RTHA with either modular (*n* = 130) or monobloc prosthesis (*n* = 129) was executed. A total of 186 cases, including 93 of each device, were finally analyzed during a mean follow-up period of 9.1 (0.5–23.1) years. The time-dependent risk of PPF was calculated using a Kaplan–Meier analysis. **Results:** The cumulative risk for PPF of the whole cohort was 5.7% (95% CI: 1.7–9.8%) at 23.1 years, for the modular device, 13.0% (95% CI: 0–26.0%) after 13.7 years and the monobloc stem, 3.4% (95% CI: 0–7.1%) after 23.1 years, without a significant difference between the two designs (log-rank *p* = 0.1922). All eight fractures occurred in women and there was one collapse of the fracture after open reduction and internal fixation. The cumulative risk was 10.1% (95% CI: 3.1–17.1%) at 23.1 years compared to 0% after 21.4 years in men (log-rank *p* = 0.0117). Diabetes was significantly associated with the presence of PPF during follow-up (non-diabetic, 4.4% (95% CI: 0–8.2%) after 21.3 years vs. diabetic, 16.6% (96% CI: 0–34.5%) after 13.3 years; log-rank *p* = 0.0066). Longer reconstructions showed also a significantly higher fracture risk (equal or longer than median implant length vs. shorter; 10.5% (95% CI: 3.1–17.1%) after 21.4 years vs. 1.0% (95% CI: 0–3.1%) after 23.1 years; log-rank *p* = 0.0276) but did not correlate with a preoperative defect situation. **Conclusions:** The cumulative risk for PPF after femoral revision with tapered and fluted devices is a relevant failure reason in this mid- to long-term investigation. There was no difference between the monobloc stem or modular implant. Women and diabetics are at risk, and the choice of a longer implant than necessary is neither prophylactical for PPF nor useful in the case of the operative treatment of a PPF after femoral RTHA with these revision devices.

## 1. Introduction

Periprosthetic fracture (PPF) represents a serious complication after total hip arthroplasty (THA). The incidence for revision due to periprosthetic fracture of 0.07% after cemented and of 0.4% after cementless primary THA is relatively low within the first two years compared to other main failure reasons like aseptic loosening or infection [1]. Nevertheless, it is increasing with time, with cumulative risk at 20 and 22 years being between 2.1% for cemented and 7.7% up to 9.4% for cementless primary THAs. This rate is at least comparable to aseptic loosening with respect to long-term follow-up studies [2,3]. Risk factors like gender, age and osteoporosis have been described, and cemented fixation seems to be preventive in primary THA [4].

Although many principle considerations may be transferable, less data are available focusing on PPF after revision THA (RTHA). A risk of 11% at 20 years for cemented and uncemented stems is published, but patient- and implant-related risk factors are less investigated [5]. RTHA cementless fixation, especially with tapered, fluted stems, shows favorable mid- and long-term results compared to cemented femoral revision [6,7]. The preventive factor of cementing with respect to PPF may be reversed by a significantly higher loosening rate of cemented stems after RTHA [8]. Due to this finding, cementless femoral revision is favored by the majority of surgeons, but long-term complications like PPF have to be taken into account. Its treatment is time-consuming, expensive and not infrequently associated with limited functional outcome.

To our knowledge, epidemiological data with respect to postoperative PPF after cementless femoral RTHA with a tapered and fluted design in a relevant number of patients and a mid- to long-term follow-up have not been published so far. PSA of two cohorts after RTHA treatment with either monobloc or modular implants was performed to obtain one cohort for the analysis of general risks and to limit potential bias due to the study protocols being retrospective analyses.

The overall risk assessment of PPF may help to assess the different failure modes after RTHA during follow-up. As the diameter or length of the device and resulting reconstruction length may not only be dependent on the underlying defect situation, the investigation of these parameters regarding PPF may be interesting. Modular implants may also have other specific complications aside from the breakage of taper junctions. Enhanced stiffness around the connection may affect the risk of PPF in the long run. Therefore, comparison of the two designs is self-evident and may help to facilitate the choice of an adequate implant and surgical technique for femoral RTHA.

## 2. Patients and Methods

### 2.1. Inclusion Criteria, Methods

**Figure 1:**
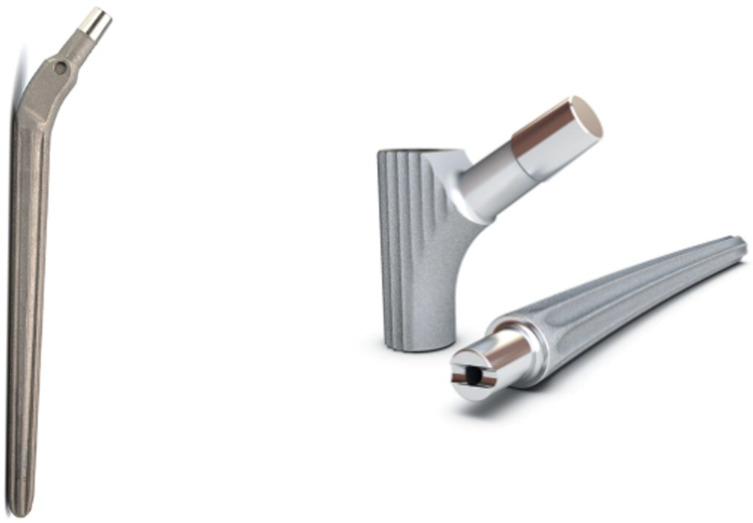
Wagner SL^®^ revision device, 2nd generation (**left**), and modular MRP^®^ (**right**).

The current retrospective study is based on two consecutive groups. In one group, the modular MRP^®^ stem (Peter Brehm, Weissendorf, Germany) and in the other group, the monobloc Wagner SL^®^ revision stem (Zimmer Biomet, Warsaw, IN, USA) was implanted (Figure 1). Design-related stem specifications are given in Table 1. Two surgeons at two different centers were involved in this investigation, and the retrospective study took place over 24 years. The relevant basic data of the patients and operations are listed in Table 2.

Only postoperative PPFs were analyzed with evidence of a stable integrated stem without loosening or subsidence. Therefore, a follow-up of at least five weeks was found to be adequate because sufficient bony ingrowth of the implant with secondary stability was assumed at that time. As (low-grade) infection may negatively influence or even prevent bony integration, all infected cases were excluded. All cases with aseptic loosening and/or subsidence without secondary stabilization and/or instability were also excluded from the study. In one case (Wagner SL cohort, WSLG), an ingrown stem but joint instability due to malpositioning of stem and cup with consecutive RTHA was present (Table 2). One patient was lost to follow-up in the MRP group (MRPG) after 33 days because the patient was from abroad and no follow-up data were available. All abovementioned and excluded patients, especially cases with the presence of late infection or loosening, had no PPF until the latest follow-up and/or revision. Information from all other revisions in both groups was available, including patients who died during follow-up. Data of these patients were included until the latest follow-up.

Basic variables of both cohorts were analyzed. Sex was normally distributed in both cohorts, but age, body mass index (BMI) and preoperative defect classification according to Paprosky [9] were not. Non-parametric testing revealed a significant difference in both groups with regard to age and defect and a tendency of the variable ‘BMI’. As it is a retrospective investigation without randomization of the patients, a propensity score analysis (PSA) (R-studio, Ver. 2023.06.01 Built 524, R Ver. 4.3.1; R Foundation for Statistical Computing, Vienna, Austria) of the two cohorts was therefore performed to reduce the bias of confounding variables. Matching of the parameters age, BMI and preoperative bone defect was executed. There were three patients with PPF among the excluded patients after matching (two in the WSLG and one in the MRPG). Further statistical analysis was performed with JMP 10 for Mac (SAS Institute Inc., Cary, NC, USA). A time to event analysis was executed using the Kaplan–Meier method with postoperative PPF as the failure criterion. Potential influencing factors like sex, age, side and body mass index (BMI), as well as radiological parameters, were investigated. The *p*-value for comparing survival curves was calculated with the log-rank-test. A *p*-value of ≤0.05 was considered significant. A 95% confidence interval was added to all survivorship data. Correlations between a continuous and/or discrete variable were tested by Student’s T-, Paired T- or Chi-square test depending on the underlying empirical distribution. All tests were two-sided and *p* ≤ 0.05 was considered significant.

All radiological evaluations were performed by two of the authors (P.B., O.B.). Bone defect classification was evaluated according to the Paprosky system [9]. The Vancouver classification system was used for description of the periprosthetic fracture [10]. Other parameters like the length of the implant and/or reconstruction and diameter of the implant were observed. To identify and exclude patients with loosening and/or subsidence according to the criteria above, further radiological evaluation was performed, including a migration analysis according to Callaghan et al. [11].

### 2.2. Surgical Technique and Postoperative Care

The implant beds for the Wagner SL stem with a straight design were prepared by corresponding reamers of the system for all available lengths. The 140 mm distal anchoring piece of the MRP device is only available as a straight model, and the 200 mm option as a straight and curved model. All longer stems are curved. For straight variants, preparation of the femoral canal was performed with corresponding reamers of the system. The preparation of all bent options was performed with flexible reamers. For the used implants, the mean diameters and reconstruction lengths including the complete device with or without the femoral head are given in Table 2.

The use of massive and/or morselized bone transplants as auto- and/or allografts for defect reconstruction is shown in Table 2. Autografts were gained from the cup during reaming in cases with complete exchange of the THA.

Two days postoperatively, patients started with physiotherapy and were mobilized. Partial weight bearing with 20 kg bodyweight was scheduled for six weeks. Afterwards, increasing weight bearing stepwise by 10–20 kg per week after X-ray control followed until full weight bearing was achieved. Active and passive exercises of hip motion were restricted to 60° of flexion for six weeks. Patients not able to achieve partial weight bearing were initially mobilized by wheelchair and started walking with full weight bearing after six weeks.

## 3. Results

### 3.1. Data Collection

Mean follow-up periods and the number of patients who died during follow-up, including the mean duration until death of the whole cohort and its two groups, are given in Table 3.

### 3.2. Periprosthetic Fractures

During follow-up, five periprosthetic fractures in the MRPG and three in the WSLG occurred (Table 4). All fractures happened after a low-impact trauma like stumbling at home and were located near the tip of the prostheses. Fracture classification according to the Vancouver system is presented in Table 4 [10]. All patients in the MRPG showed a displaced fracture and were treated by open reduction and internal fixation by plate (ORIF). In the WSLG, two patients with fractures were treated by ORIF, whereas in the other patient, conservative treatment of an incomplete and undisplaced fracture was performed by bracing over a period of six weeks. The non-union and collapse of one fracture in the MRPG occurred after closed reposition and internal fixation by plate. All other fractures healed and the stems were stable during further follow-up.

### 3.3. Survivorship Analysis, Risk Factors for PPF and Radiological Findings

**Figure 2:**
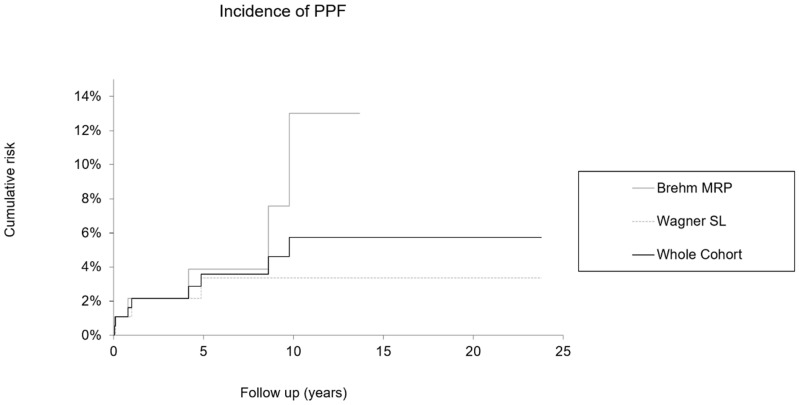
Risk for PPF, MRPG, WSLG and whole cohort (without 95% CI).

Cumulative risk for PPF was calculated for the Wagner SL, the MRP system and for the whole cohort of all 186 cases (Figure 2, Table 3). The risk of modular stems was higher compared to the Wagner monobloc stem (13.0% vs. 3.4%), although the Wagner SL cohort had a longer follow-up (Table 3). Nevertheless, the risk for occurrence of a PPF between the two groups was not significantly different (log-rank test: *p* = 0.1922).

**Figure 3:**
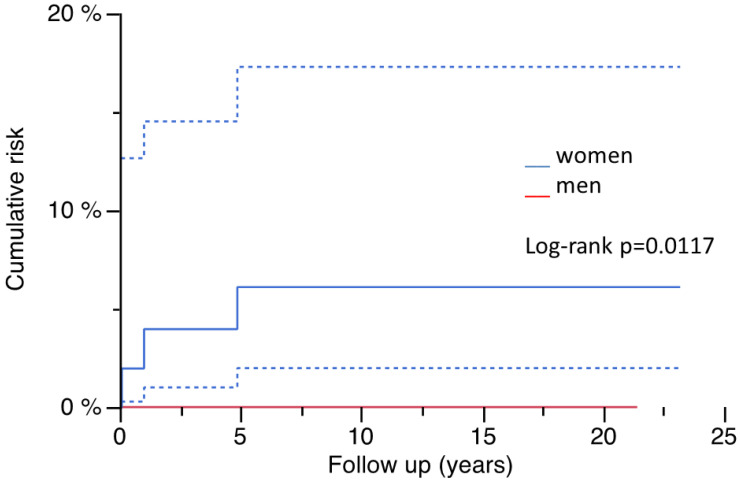
Risk for PPF and gender (95% CI).

Of the analyzed basic factors after matching by PSA, only sex had a significant impact on the occurrence of PPF in the whole cohort (Figure 3) and the MRPG, but not in the WSLG (Table 3), although all patients presenting with a PPF were female (Table 4). Pearson’s chi-square-test revealed the same correlation of sex and occurrence of PPF (whole cohort: *p* = 0.0021; WSLG: *p* = 0.0548; MRPG: *p* = 0.0176).

**Figure 4:**
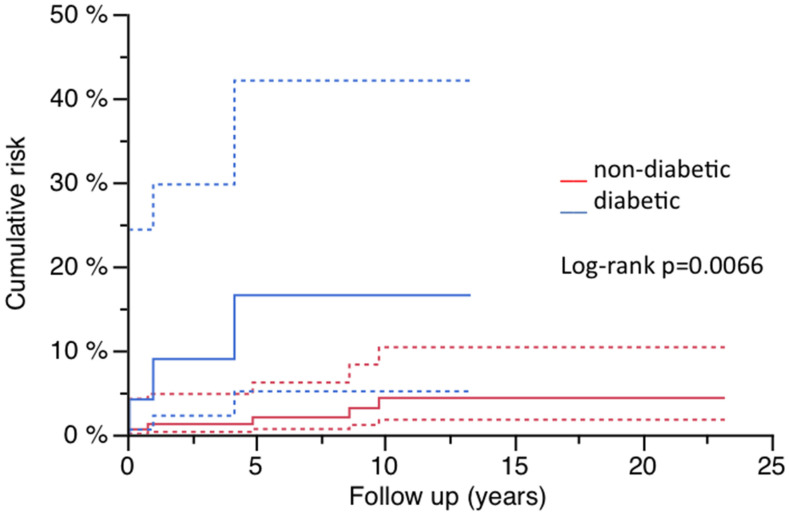
Risk for PPF and diabetes (95% CI).

Diabetics were also at a significantly higher risk for PPF during follow-up in the whole cohort and WSLG (Figure 4, Table 3). The same relation was shown by Pearson’s chi-square-testing (whole cohort: *p* = 0.0339; WSLG: *p* = 0.0015; MRPG: *p* = 0.7505).

**Figure 5:**
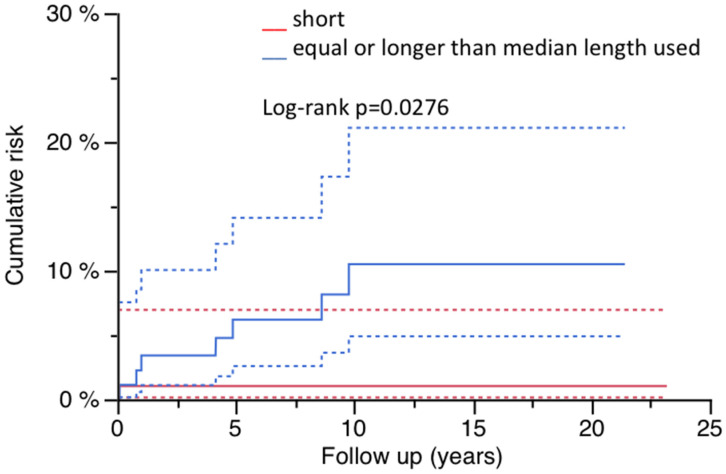
Risk for PPF and implant length (95% CI).

Regarding the whole cohort, PPF occurred significantly more frequently with stems equal or longer than the calculated median length (*n* = 7) compared to shorter stems (*n* = 1; Figure 5). Examination within the WSLG and MRPG cohort by the Kaplan–Meier method revealed no significant influence of implant length on fracture risk, although all fractures occurred in patients treated with longer implants in the WSLG, and all but one in the MRPG (Table 3). However, Pearson’s chi-square testing showed a significant impact in the whole cohort and WSLG (*p* = 0.0158 and *p* = 0.0375).

## 4. Discussion

### 4.1. Background and Rationale

The absolute number of PPFs after primary THA is estimated to be from about 0.6% to 6.9% depending on implant design, surgical approach, indication for primary THA, aseptic loosening, osteoporosis and age [12]. In the Swedish Hip Arthroplasty Register, PPF constituted up to 13% of reoperations after primary THA and 5% after former RTHA in 2020 [13]. Nevertheless, time-dependent data using a Kaplan-Meier or life-table analysis to compare PPF after RTHA with other failure reasons in a mid- to long-term follow-up is limited.

Age- and gender-related problems like osteoporosis or implant-dependent impact may be more relevant after RTHA because the mean age of those patients is inevitably higher. Additional factors with impacts on the intraoperative or postoperative outcome of surgical treatment of PPF after RTHA have to be taken into account in this context. The adequate choice of surgical algorithm, including the approach, osteosynthesis and/or suitable endoprosthetic device, as well as effective but conservative anesthesia, is mandatory for the successful treatment of these elderly patients. In the case of spinal anesthesia as one option, intraoperative adverse hemodynamic reactions can be minimized without compromise on comfort and sensorimotor effect [14]. Therefore, the treatment of these patients determines a multidisciplinary setup experienced in geriatric traumatology. Independently, the surgical treatment of PPF even with implanted primary hip stems shows high complication rates and needs further research with respect to prevention as well as surgical treatment [15,16].

### 4.2. Cumulative Risk of PPF and Predicting Factors

A cumulative risk of the whole cohort of 5.7% at 23.1 years is concerning and, compared to some studies, higher than the cumulative risk for aseptic loosening or even infection after cementless femoral revision using these implants within this follow-up period [17,18,19,20]. Nevertheless, the cumulative risk for PPF is lower compared to cementless primary THA [2,3]. PPFs of the modular MRP stem appeared more frequently compared to the Wagner SL monobloc device (13.0% vs. 3.4%), even though the WSLG had a longer follow-up. This finding is due to the two late PPFs in the MRPG more than eight years postoperatively. Although direct correlation revealed no significant difference between the two groups, the negative influence may have resulted from the modular buildup and lower elasticity of the modular stem in the long run. According to manufacturers’ data (Zimmer Biomet, Warsaw, IN, USA; EMEA Winterthur, Switzerland) comparing the Revitan^®^ and Wagner SL^®^ device, modularity causes a lower elasticity modulus around the junction area. Bony regeneration was obvious with the Wagner Monobloc stem even in higher preoperative defect situations reconstructing proximally from the anchoring area [18,21]. Restoration proximal to the junction area after modular RTHA was rarely seen in this MRPG cohort, and stress-shielding with the limited anchoring length of the whole construct may have been a result. A rising risk of PPFs, as well as the breakage of junctions, may be a problem in the long run after revision with modularly built devices, but long-term investigations are missing so far.

Age, gender and/or the presence of osteoporosis are factors influencing the risk for PPF after primary THA [4]. The cumulative risk of PPF in women was significantly higher compared to men in the whole cohort (10.1% vs. 0%) as well as in the modular MRPG (22.5% vs. 0%). Although all fractures occurred in women, the relation was not significant in the WSLG (6.1% vs. 0%). As diabetes influences osteoporosis, a significant impact was shown on the whole cohort (16.5% vs. 4.4%) and the WSLG (20.0% vs. 12.8%), but not in the MRPG (20.0% vs. 12.5%). Diagnosis of osteoporosis as daily routine in patients presenting with PPF is currently missing but should be carried out after the treatment of the fracture as the assessment of bone quality is mandatory to prevent further complications. Cemented fixation in the case of missing cancellous bone or the presence of fracture gaps is obsolete, and cementless fixation remains the method of choice when performing femoral RHTA. Consecutively, there are limited options with regard to surgical treatment, choice of osteosynthesis or endoprosthetic device. In summary, the prevention of falls and of osteoporosis or its medical treatment should be performed and may have a positive influence on the occurrence of PPF, especially in patients who are at risk [22].

Patients with implants equal to or longer than the determined median length of the two implants showed a significantly higher time-dependent risk of PPF in the whole cohort (10.5% after 21.4 years vs. 1.0% after 23.1 years; log-rank test: *p* = 0.0276), but this was only by trend in the WSLG and not significant in the MRPG. All but one fractures occurred with stems longer than the median length. The underlying defect according to the system described by Paprosky may play a role but could not be confirmed statistically. This may be due to the descriptive nature of the system with a certain general but sometimes limited value with regard to the individual biomechanical situation of the (deficient) femoral bone and loading capacity. Longer implants are intra-operatively used and accepted but often not necessary according to the underlying biomechanical situation, including the bone defect with or without additional breaking points and general bone quality. The surgical approach and use of bone grafts and/or additional osteosynthesis may also affect the choice of the implant length.

In contrast to the findings in primary THA, especially with cemented stems, using a longer implant may not be preventive of the occurrence of a PPF, particularly with the presence of osteoporosis. As long as necessary and as short as possible remains the primary criterion for selection of the stem length in RTHA. This may also help to address occurring fractures as more bone substance off the implant bed is available to perform ORIF and/or exchange of the stem. Independently, preoperative planning and intraoperative adaptation is mandatory and more standardized algorithms are necessary.

### 4.3. Limitations

There are differences with respect to the two stem designs used. To our knowledge, no stem device is available as a monobloc or modular variant with completely identic design characteristics except the junction area of the modular implant. There may be potential influences due to the (minimal) differences in the stem designs. Nevertheless, once bony integrated, this factor may be insignificant. On the other hand, differences in the design with significant influences on the fracture risk may be of high interest and helpful in choosing a suitable implant and developing standardized treatment protocols based on pre- or intraoperative findings and specific factors of the patient.

The data were collected from only two centers and during different time periods. Nevertheless, both units were experienced in RTHA, as the number of involved patients proves. To minimize the potential influences of the patients being collected at two different centers, propensity score matching was performed. Although matching by PSA may have reduced the bias, a limitation remains due to the study protocol being a retrospective analysis. Overall, two consecutive series with 93 cases each after matching and a total of 186 patients seem to be adequate to answer the questions asked.

## 5. Conclusions

Gender and diabetics, as influencing factors for osteoporosis, are also predictive for the occurrence of PPF during follow-up after cementless femoral RTHA using a tapered and fluted design. The design itself, whether modularly built or as a monobloc implant, creates no difference to the appearance of PPF in this study. In addition, the use of longer stems than necessary is not preventive for PPF. Overall, the cumulative risk of PPF is relatively high in this mid- to long-term follow-up.

## Figures and Tables

**Table 1:** Design specifications.

	Wagner SL *	MRP Brehm ^#^
**Length in mm**	190	140
	225	200
	265	260
	305	320
	345	
	385	
**Diameter in mm**	190: 14–20	140 and 200: 13–30
	225: 14–22	260 and 320: 11–29
	265 to 385: 14–25	
**Offset: CCD angle in degree**	145	Standard neck piece: 130
		Lateralized neck piece: 123.5
**Neck piece: length in mm**	-	Short = 50
		Medium = 60
		Long = 70
**Lengthening piece in mm**	-	30
**Longitudinal ridges**	8	8
**Lead angle in degree: anchoring area/distal body**	2	140 mm: 2.5
		200 mm: 1.8
		260 mm: 0.5
		320 mm: 0.4

* Wagner SL: length measured from cone to tip. A lead angle of 2° exists over distal 120 mm to the tip and the nominal diameter is measured 123 mm proximally from tip. ^#^ MRP Brehm: the length of the distal anchoring body is given. Nominal diameter for the 140 and 200 mm piece is measured at two-thirds of the length proximally from tip. The nominal diameter of the 260 and 320 mm device is measured at the tip.

**Table 2:** Basic data of the patients and index operation.

	Wagner SL	MRP Brehm	Whole Cohort
**Original cohorts:**	129	130	259
**Excluded patients due to:**			
**Infection**	4	7	11
**Aseptic loosening**	2	5	7
**Instability**	1	0	1
**Intraoperative PPF** ^#^	1	0	1
**Lost to follow-up**	0	1	1
**PSA related**	28	24	52
**Included no. of patients**	93	93	186
**Implanted between (year)**	1989–1996	2003–2009	1989–2009
**Indication:**			
**Aseptic loosening**	73	47	120
**Septic two-stage revision**	3	35	38
**Septic one-stage revision**	9	0	9
**Periprosthetic fracture**	8	11	19
**Surgeons involved**	7	8	15
**Sex (w/m)**	51/42	54/39	105/81
**Operated side (r/L)**	48/45	46/47	94/92
**BMI in kg/m^2^**	26.8 (18.0–36.3)	27.0 (18.0–39.5)	26.9 (18.0–39.5)
**Mean age at surgery (range) in years**	65.6 (36.7–86.3)	68.2 (38.9–85.4)	66.9 (36.7–86.3)
**No. of used stems/distal anchoring bodies**	190 mm: 1	140 mm: 29	
	225 mm: 13	200 mm: 58 (incl. 2 straight stems)	
	265 mm: 33	260 mm: 6	
	305 mm: 38		
	345 mm: 7		
	385 mm: 1		
**Neck piece (standard/lateralized)**	-		
**Short (50 mm)**		44 (28/16)	
**Medium (60 mm)**		31 (21/10)	
**Long (70 mm)**		18 (4/14)	
**Lengthening piece (30 mm)**	-	30	
Mean reconstruction length (range)/median *			
**Without femoral head**	282.3 (190–385)/265	247.8 (190–320)/250	267.4 (190–385)/265
**With femoral head**	281.9 (186–389)/269	248.1 (186–320)/254	269.0 (186–389)/265
Mean stem diameter (range)/median *	16.5 (14–22)/16	17.0 (13–23)/16	16.7 (13–23)/16
**Surgical approach:**			
**Transfemoral**	47	15	62
**Transtrochanteric**	1	0	1
**Hardinge/transgluteal**	34	78	112
**Posterior**	11	0	11
**Preoperative bone defect (Paprosky)**			
**Grade 1**	16	13	29
**Grade 2**	59	62	121
**Grade 3A**	4	4	8
**Grade 3B**	10	10	20
**Grade 4**	4	4	8
**Bone transplant at the femur:**			
**Total**	35	44	79
**Autogeneous**	8	6	14
**Allogeneous**	25	38	63
**Both**	2	0	2
**Morselized**	28	40	68
**Bulk/strut graft**	1	2	3
**Both**	6	2	8

**^#^** and insufficient osteosynthesis; * all specifications in millimeters.

**Table 3:** Results.

	Wagner SL	MRP Brehm	Whole Cohort
Mean follow-up (range) in years *	12.7 (0.8–23.1)	6.0 (0.5–13.7)	9.1 (0.5–23.1)
**Death during follow-up**	42	9	51
**Mean follow-up of deceased patients (range) in years**	9.5 (0.8–21.6)	2.9 (0.5–7.8)	8.3 (0.5–21.6)
**PPF during follow-up**	3	5	8
**Follow-up until PPF (range) in years**	2.0 (0.1–4.9)	4.3 (0.1–9.8)	3.4 (0.1–9.8)
**Overall risk of PPF (95% CI) in %**	3.4 (0–7.1) after 23.1 years	13.0 (0–26.0) after 13.7 years	5.7 (1.7–9.8) after 23.1 years (log-rank test: *p* = 0.1922) ^#^
**Risk of PPF (95% CI) and sex in %: female vs. male**	6.1 (4.5–17.0) after 23.1 years vs. 0 after 21.4 years (log-rank *p* = 0.1135)	22.5 (1.4–43.5) after 13.7 years vs. 0 after 13.5 years (log-rank *p* = 0.0487)	10.1 (3.1–17.1) after 23.1 years vs. 0 after 21.4 years (log-rank *p* = 0.0117)
**Risk of PPF (95% CI) and diabetes in %: non-diabetic vs. diabetic**	12.8 (10.3–15.3) after 23.1 years vs. 20.0 (0–44.8) after 13.3 years (log-rank *p* = 0.0004)	12.5 (0–26.1) after 13.7 years vs. 20.0 (0–55.1) after 10.1 years (log-rank *p* = 0.4884	4.4 (0–8.2) after 23.1 years vs. 16.6 (0–34.5) after 13.3 years (log-rank *p* = 0.0066)
**Risk of PPF (95% CI) and reconstruction length in %: longer than median length vs. shorter or equal**	6.8 (0–14.3) after 21.4 years vs. 0 after 21.4 years (log-rank *p* = 0.0741)	22.9 (0–46.5) after 13.7 years vs. 2 (0–5.9) after 11.6 years (log-rank *p* = 0.1806)	10.5 (2.8–18.2) after 21.4 years vs. 1.0 (0–3.1) after 23.1 years (log-rank *p* = 0.0276)

* without failures due to periprosthetic fracture; ^#^ risk of PPF between the two groups (MRP vs. Wagner).

**Table 4:** Periprosthetic fractures.

Patient, Age at Surgery (ys.), Gender	System	Indication ^#^	Bone Defect Paprosky [9]	Approach ^§^	BMI (kg/m^2^)	Diabetes (y/n)	PPF Postop. (ys.)	Vancouver Classifi-Cation	Conservative (C) vs. Operative Therapy (O)	Stem/ Reconstruction Length/Diameter (mm) *
77, f.	MRP	TSR	4	TG	20.8	y	4.2	C	O (ORIF by Plate)	290/14
68, f.	MRP	AL	2B	TG	22.5	n	0.8	C	O (ORIF by Plate, Liss **^©^**)	250/18
73 ^+^, f.	MRP	AL	3B	TG	34.4	n	8.6	C	O (MIS by Plate, VA **^©^**)	290/21
75, f.	MRP	AL	2B	TG	25.9	n	9.8	C	O (ORIF by Plate, VA **^©^**)	250/19
76, f.	MRP	AL	2A	TG	31.8	n	0.1	B1/C	O (ORIF by Plate, Liss **^©^**)	240/22
57, f.	WSL	AL	2A	TG	35	y	0.1	B1/C	O (ORIF by Plate, Condylar)	305/17
72, f.	WSL	AL	3B	P	29	n	4.9	C	O (ORIF by Plate, Condylar)	305/14
79, f.	WSL	AL	3B	TF	22	y	1.0	B1/C	C	305/16

^#^ Two-stage revision (TSR); aseptic loosening (AL); periprosthetic fracture (PPF); ^§^ transgluteal-lateral/Hardinge (TG); transfemoral (TF), posterior (P); * stem length of the Wagner SL: complete length; stem length of the MRP system: distal anchoring device and neck, all stems were curved; ^+^ closed reduction and minimally invasive osteosynthesis. This patient had a former supracondylar fracture many years before RTHA. Collapse of the fracture with non-union within two months.

## Data Availability

The datasets presented in this article are not readily available because the data are part of an ongoing study. Requests to access the datasets should be directed to the corresponding author.
